# Supplementary material for: Effects of Orally Delivered Double-Stranded RNA of Trehalose-6-Phosphate Synthase on the Population of Frankliniella occidentalis
Source: Insects. 2025 Jun 10;16(6):614. doi: 10.3390/insects16060614 (PMC12193368; doi:10.3390/insects16060614)
Supplement: Supplementary file 1 [file insects-16-00614-s001.zip › insects-3655619-supplementary.pdf]

Supplementary Materials

Table S1. Primer synthesis sequence information

Name	Sequence(5′- 3′)	Purpose
dsTPS_F	taatacgactcactatagggttgattgctgccaaagacgc	Synthesis of dsRNA
dsTPS_R	taatacgactcactataggggccacagcagcatctctat	
dsEGFP_F	taatacgactcactatagggaggagcgcaccatcttcttc	
dsEGFP_R	taatacgactcactataggggactgggtgctcaggtagtg	
TPS_qF	gacaaggttagagcagttca	qPCR
TPS_qR	gaatgaagctctatcaggca	
Actin_F	gagtccctgttccagccttc	Reference gene
Actin_R	atgtcgacgtcgcacttcat	
L4TPS-F	tggcggccgctctagattgattgctgccaaagacgct	Vector construction of L4440
L4TPS-R	cgggccccccctcgagggccacagcagcatctctataaaatg	
L4440-F	actatagggagaccggcagat	Colony PCR
L4440-R	gggaagaaagcgaaaggagc	

Table S2. Parameters, equations, and definitions used in the life table analysis

Parameter	Equation	Explanation
l_x	$l_x = \sum_{j=1}^{\beta} s_{xj}$	Age-specific survival rate, where β is the number ofstages.s_{xj}, the probability of a newborn surviving toage x and stage j.
m_x	$m_x = \frac{\sum_{j=1}^{k} s_{xj} f_{xj}}{\sum_{j=1}^{k} s_{xj}}$	Age-specific fecundity of the cohort at age x. f_{xj}, age-stagespecific fecundity.
r	$\sum_{x=0}^{\infty} e^{-r(x+1)} l_x m_x = 1$	Intrinsic rate of increase.
λ	$\lambda = e^r$	Finite rate of increase.
R_0	$R_0 = \sum_{x=0}^{\infty} l_x m_x$	Net reproduction rate.
T	$T = \frac{\ln R_0}{r}$	Mean generation time.
e_{xj}	$e_{xj} = \sum_{i=x}^{\infty} \sum_{y=j}^{k} s'_{iy}$	Age-stage life expectancy.
v_{xj}	$v_{xj} = \frac{e^{r(x+1)}}{s_{xj}} \sum_{i=x}^{\infty} e^{\ r(i+1)} \sum_{y=j}^{k} s'_{iy} f_{iy}$	Age-stage reproductive value.

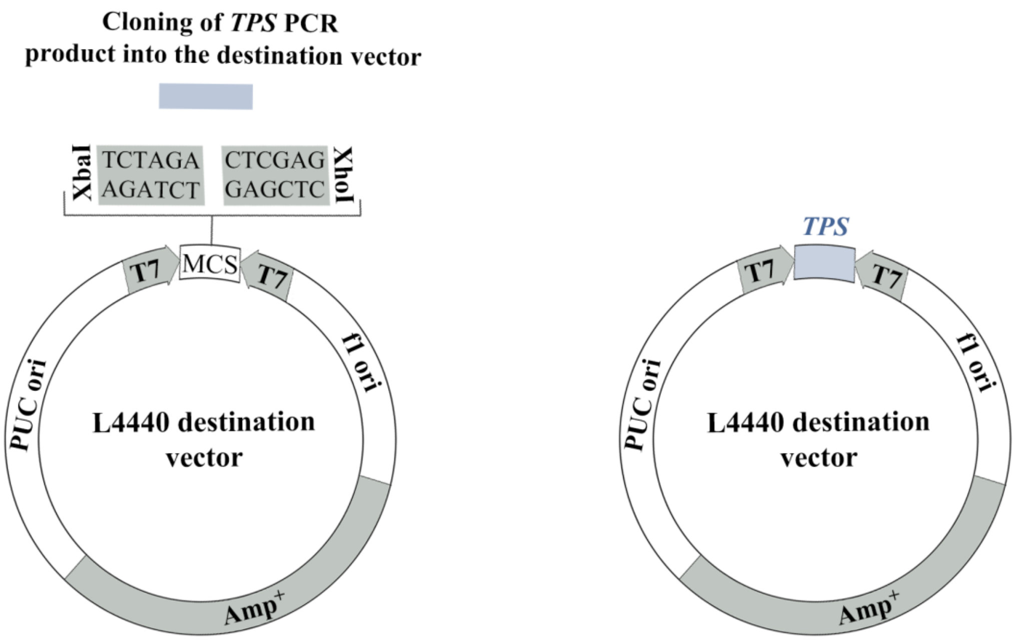

Figure S1. Schematic diagram for development of recombinant plasmids.

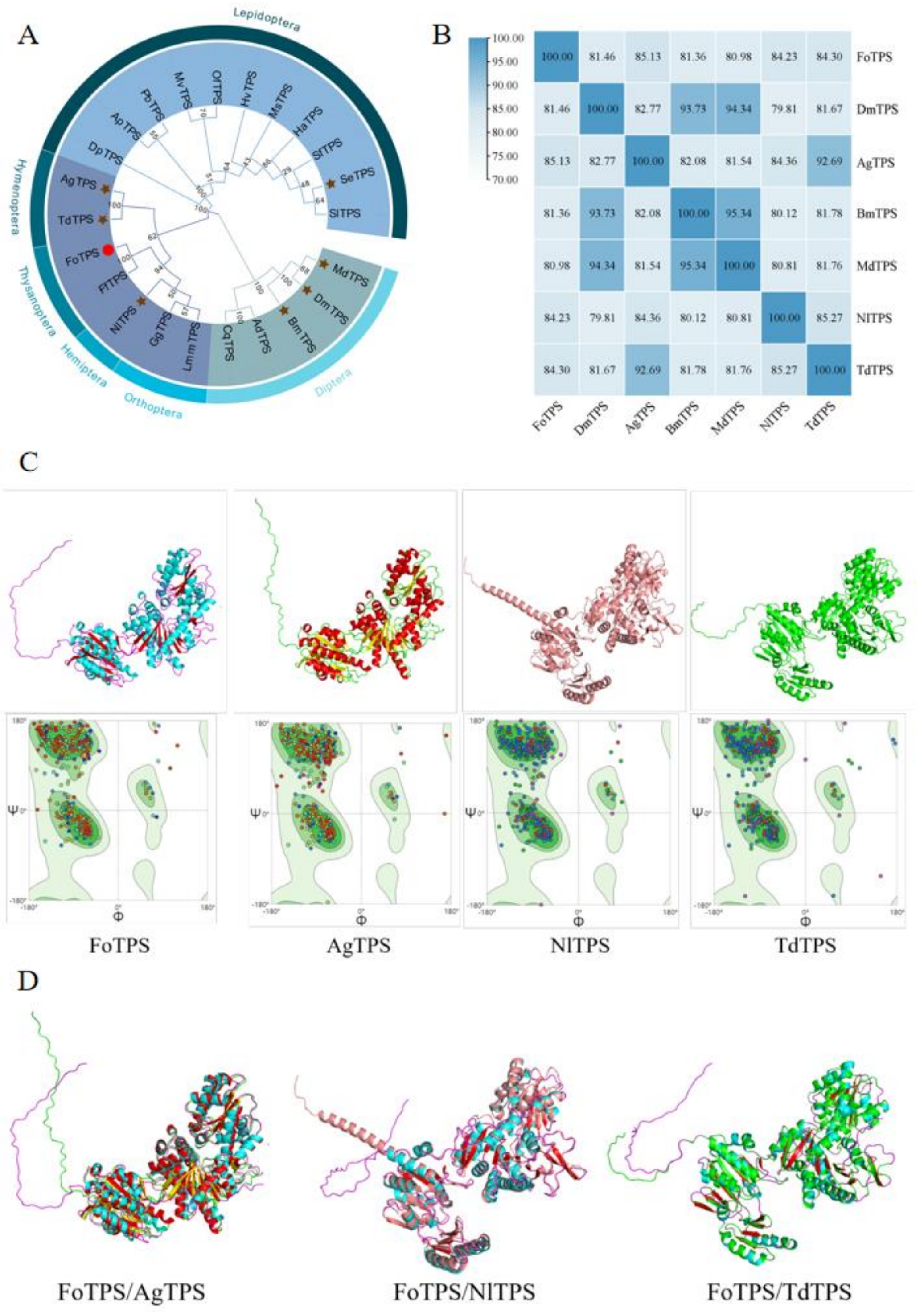

Figure S2. Phylogenetic tree, sequence similarity and three-dimensional structure of the TPSs of *Frankliniella occidentalis* and reference species. (A) Phylogenetic analysis of insect TPS proteins. Phylogenetic analysis of TPSs was based on the sequences of 11 species of Lepidoptera, 5 species of Diptera, 2 species of Hymenoptera, 2 species of Thysanoptera, 1 species of Hemiptera, 2 species of Orthoptera, and the phylogenetic

tree was constructed through the neighbor-joining (N-J) method with 1000 bootstrap replicates. FoTPS is marked with red circles and reference speciesare marked withbrownstar. (B) Sequence similarity heatmap of TPSs from 7 species, including *F. occidentalis*, *Musca domestica*, *Drosophila melanogaster*; *Trichogramma dendrolimi*, *Bactrocera minax*, *Nilaparvata lugens*, *Aphidius gifuensis*. (C) The prediction of 3D structure models include FoTPS, AgTPS, NlTPS, TdTPS, and Ramachandran plot of the corresponding model. (D) 3D structure alignments of FoTPS and AgTPS, FoTPS and NlTPS, FoTPS and TdTPS.

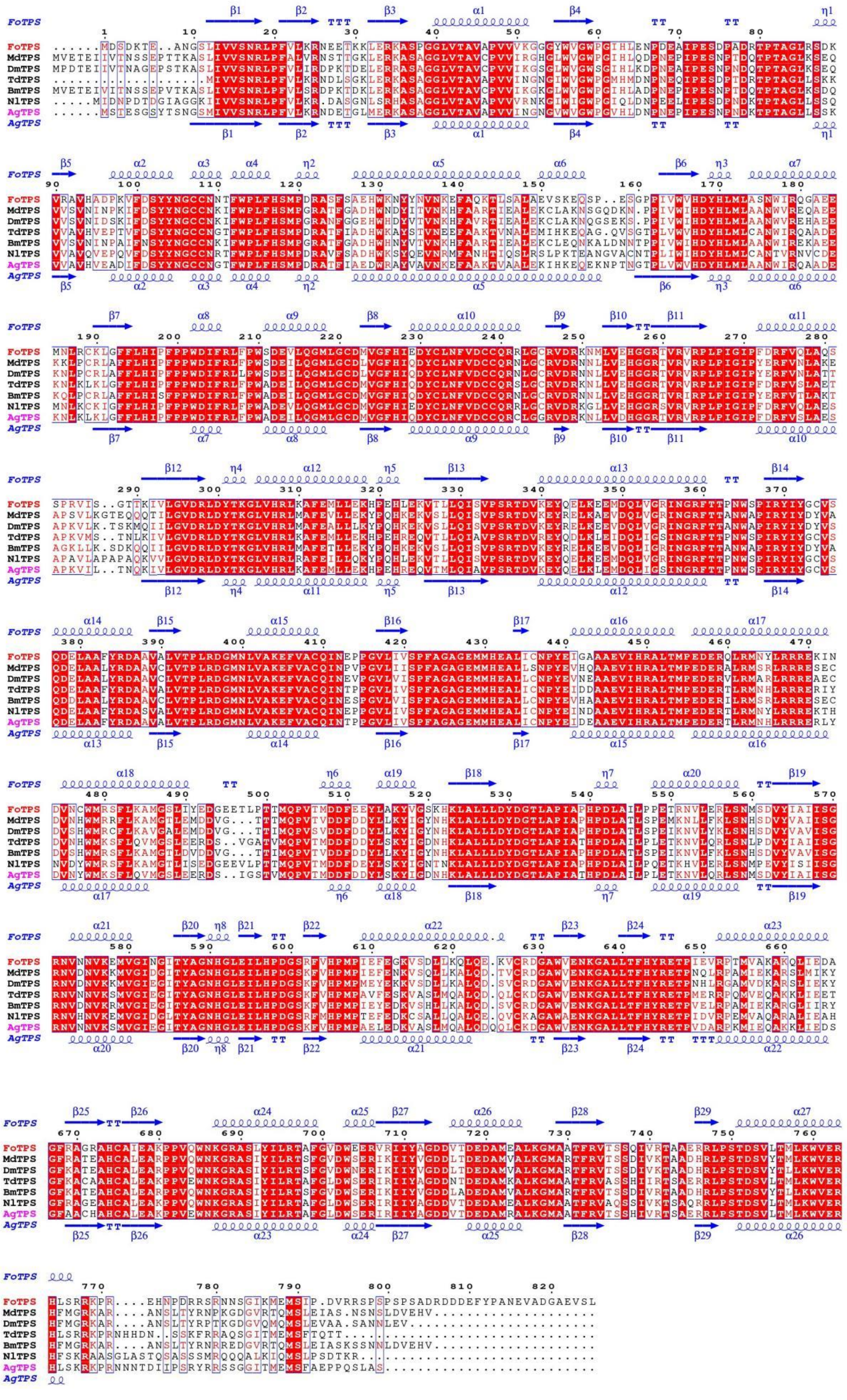

Figure S3. Secondary structure-based multiple sequence alignment of TPSs of *Frankliniella occidentalis* and reference species. A species abbreviation was provided

prior to each TPS protein name: Fo, *Frankliniella occidentalis*; Md, *Musca domestica*; Dm, *Drosophila melanogaster*; Td, *Trichogramma dendrolimi*; Bm, *Bactrocera minax*; Nl, *Nilaparvata lugens*; Ag, *Aphidius gifuensis*. The secondary structures of FoTPS and AgTPS arecolored blue, structures include α-helix, βchain, η: 3_{10}-helix, TT: strict β-turns, TTT: strict α-turns.

Table S3. GenBank assession numbers of *TPS* in different species

Gene	Species name	GenBank accession number
SeTPS	*Spodoptera exigua*	ACD01424.1
SlTPS	*Spodoptera litura*	ADA63844.1
SfTPS	*Spodoptera frugiperda*	QNS31101.1
HaTPS	*Helicoverpa armigera*	AAY87162.2
MsTPS	*Mythimna separata*	QNT61277.1
HvTPS	*Heortia vitessoides*	AYO46920.1
OfTPS	*Ostrinia furnacalis*	APD15513.1
MvTPS	*Maruca vitrata*	ATU31386.1
ApTPS	*Antheraea pernyi*	ARD05072.1
PbTPS	*Parnassius bremeri*	ATU31390.1
DpTPS	*Danaus plexippus*	OWR52345.1
DmTPS	*Drosophila melanogaster*	AAF51020.1
MdTPS	*Musca domestica*	AIC64309.1
BmTPS	*Bactrocera minax*	ANT46147.1
CqTPS	*Culex quinquefasciatus*	EDS32889.1
AdTPS	*Anopheles darlingi*	ETN66003.1
GgTPS	*Gampsocleis gratiosa*	APZ77037.1
LmmTPS	*Locusta migratoria manilensis*	ABV44614.1
NlTPS	*Nilaparvata lugens*	ACV20871.1
FoTPS	*Frankliniella occidentalis*	XP 026272691.1
FfTPS	*Frankliniella fusca*	KAK3920642.1
AgTPS	*Aphidius gifuensis*	QDL52663.2
TdTPS	*Trichogramma dendrolimi*	QUU43765.1
